# Effects of Transcranial Direct Current Stimulation (tDCS) and Exercises Treadmill on Autonomic Modulation of Hemiparetic Patients Due To Stroke—Clinic Test, Controlled, Randomized, Double-Blind

**DOI:** 10.3389/fneur.2019.01402

**Published:** 2020-01-22

**Authors:** Glauber Heinz, Katia De Angelis, Simone Dal Corso, Maria Helena Gomes De Sousa, Ariane Viana, Fernando Dos Santos, João Carlos Ferrari Corrêa, Fernanda Ishida Corrêa

**Affiliations:** ^1^Doctorate and Master Program in Rehabilitation Science of University Nove de Julho, UNINOVE, São Paulo, Brazil; ^2^Doctorate and Master Program in Medicine School of University Nove de Julho, UNINOVE, São Paulo, Brazil; ^3^Arterial Hypertension Unit, Instituto do Coração (InCor), Medical School of Universidade de São Paulo, São Paulo, Brazil

**Keywords:** stroke, autonomic modulation, transcranial direct current stimulation, exercise treadmill, hemiparetic patients

## Abstract

**Background:** After a Stroke, there is an autonomic nervous system (ANS) changes. Transcranial Direct Current Stimulation (tDCS) can promote the reorganization of the affected circuits.

**Objective:** To evaluate the effects of tDCS applied before a session of physical activity on the treadmill, in the modulation of the autonomic nervous system of post-stroke patients.

**Methodology:** Cross-over study, were randomized 12 adult hemiparetic subjects in 2 groups, Group 1 (active tDCS before exercise on the treadmill) and Group 2 (sham tDCS before exercise on the treadmill). Stimulation times were 20 min; treadmill time was 20 min. The heart rate variability (HRV) and Variability of Systolic Blood Pressure (VSBP) were evaluated for 15 min, in 3 periods (pre and post tDCS and during exercise recovery on the treadmill).

**Results:** There was no difference in the VSBP and the HRV between the groups, compared with the baseline data; however, in the intragroup analysis, the parasympathetic modulation after active tDCS increased by 18% over baseline by the RMSSD with IC 95% (−7.85 to −0.34). In group 1, the post-tDCS active and post-exercise periods presented a value of variance above baseline, indicating a better prognosis. In group 2, there was a significant reduction of 38% of Variance values (*p* = 0.003) after tDCS sham.

**Conclusion:** tDCS does not generate immediate effects on HRV and VSBP, except for intragroup comparison, which has greater participation in parasympathetic modulation in the group receiving active tDCS.

## Introduction

One of the most serious causes of stroke reoccurrence is the alteration in the Autonomic Nervous System (ANS) function that leads to a reduction of heart rate variability (HRV). This was due to the disbalance to the homeostasis of the autonomic control, based on cardiac interbeat intervals analyzed by the physiological information, which can lead to myocardial infarction and sudden death (1–3).

Damages to ANS can also affect the control of blood pressure (BP). McLaren et al. (4) conducted a case-control cross-study in which compared the autonomic function of patients that had a stroke, not-treated, with resident controls in the Community and observed that the blood pressure alteration was maintained several months after the stroke.

Although the mechanisms of this cardiovascular autonomic dysregulation have not been fully understood, several studies (3, 5–7) suggest an anatomical-functional asymmetry between the right and left cerebral hemispheres in ANS modulation activity. Dutsch et al. (8) observed that stroke survivors whose infarction occurred on the right side had increased sympathetic cardiac modulation and parasympathetic cardiac deficit. The changes after the stroke in autonomic nervous system showed that favor increased tonus sympathetic and reduced parasympathetic appear to be independent to the both hemisphere injury (1). Macey et al. (7) and Al-Qudah et al. (3) reported that an important area in autonomic control is the insular cortex, with the right lobe insular cortex related to sympathetic modulation and the insular cortex of left lobe responsible for parasympathetic modulation. The insular cortex is located topographically in the neocortex region below the parietal, temporal and frontal cortex (5).

Upon the importance of the ANS in cardiovascular control, some studies have researched the effects of Transcranial Direct Current Stimulation (tDCS) over the left temporal cortex in the ANS modulation. One of them is the study from Montenegro et al. (6) that applied the tDCS in healthy athlete and none athlete individuals. They observed a reduction in the improved HRV in rest and quicker recovery from the exercise for athlete individuals. Another study is from Okano et al. (9) that observed a better parasympathetic action and decrease in the effort sensation and fatigue of healthy individuals during the exercise with cycle ergometer. Cogiamanian et al. (10) observed that the modulation of cortical excitability by tDCS might be used as a possible tool for human artery hypertension treatment.

The use of tDCS in individuals with stroke is safe and may be used during the rehabilitation to promote the reorganization of circuits affected by the lesion (11). However, its effects were not researched in the modulation of ANS by the temporal area in these individuals.

With the increase in surviving patients from stroke events, it is important to expand the comprehension of the lesions' chronic effects and that interferences in this sense, when positive, will enable better prognosis of cardiac autonomic function, reducing the possibility of a stroke relapse and sudden death and providing also better quality of daily living.

Therefore, the main objective of the study was to evaluate the HRV and the Variability of Systolic Blood Pressure (VSBP) in hemiparetic patients due to stroke in basal conditions (rest), immediately after the application of active tDCS and sham (pre-exercise) and during the exercise recovery on the treadmill. As a secondary outcome, the performance of the exercise, and perception of stress was evaluated, with the cardiac parameters (cardiac rate, modified Borg for dyspnea and fatigue, distance traveled).

## Methodology

This study consists of an original cross-sectional, randomized, controlled, double-blind trial study of 12 adult hemiparetic stroke patients. The study followed the norms set by the Consort.

Survey participants included those with stroke hemiparesis for more than 6 months (12); from 21 to 74 years old; with mild or moderate lower limb motor impairment [between 20 and 31 points on the Fugl-Meyer lower limb scale (13)]; which presented comfortable walking speed between 0.3 and 1.15 m/s (14); walk at least 50 meters (15) without difficulty; agreeing to sign a consent and informed form.

Those with cognitive impairment (Mini-Mental State Examination-MMSE) with a score below 24 points (16) were excluded so that they could understand the study protocol; visual alteration that would prevent the execution of the protocol; severe heart problems (congestive heart failure, angina, peripheral vascular disease). Use of pacemaker; contraindications to the use of tDCS (brain metal clip implant near the region to be stimulated, history of recurrent seizure, recurrent epilepsy, brain tumors, brain pacemaker, and opening in the skullcap); women who had an irregular menstrual cycle or who were in the menstrual cycle during the evaluation; without medical clearance for treadmill exercise testing.

This study was performed at University Nove de Julho at unit Vergueiro and approved by Ethics Committee of University Nove de Julho (protocol CAAE: 57314716.7.0000.5511), São Paulo, Brazil and registered in Clinical Trial (NCT02956096) https://clinicaltrials.gov/ct2/show/NCT02956096.

## Intervention

**Figure 1:**
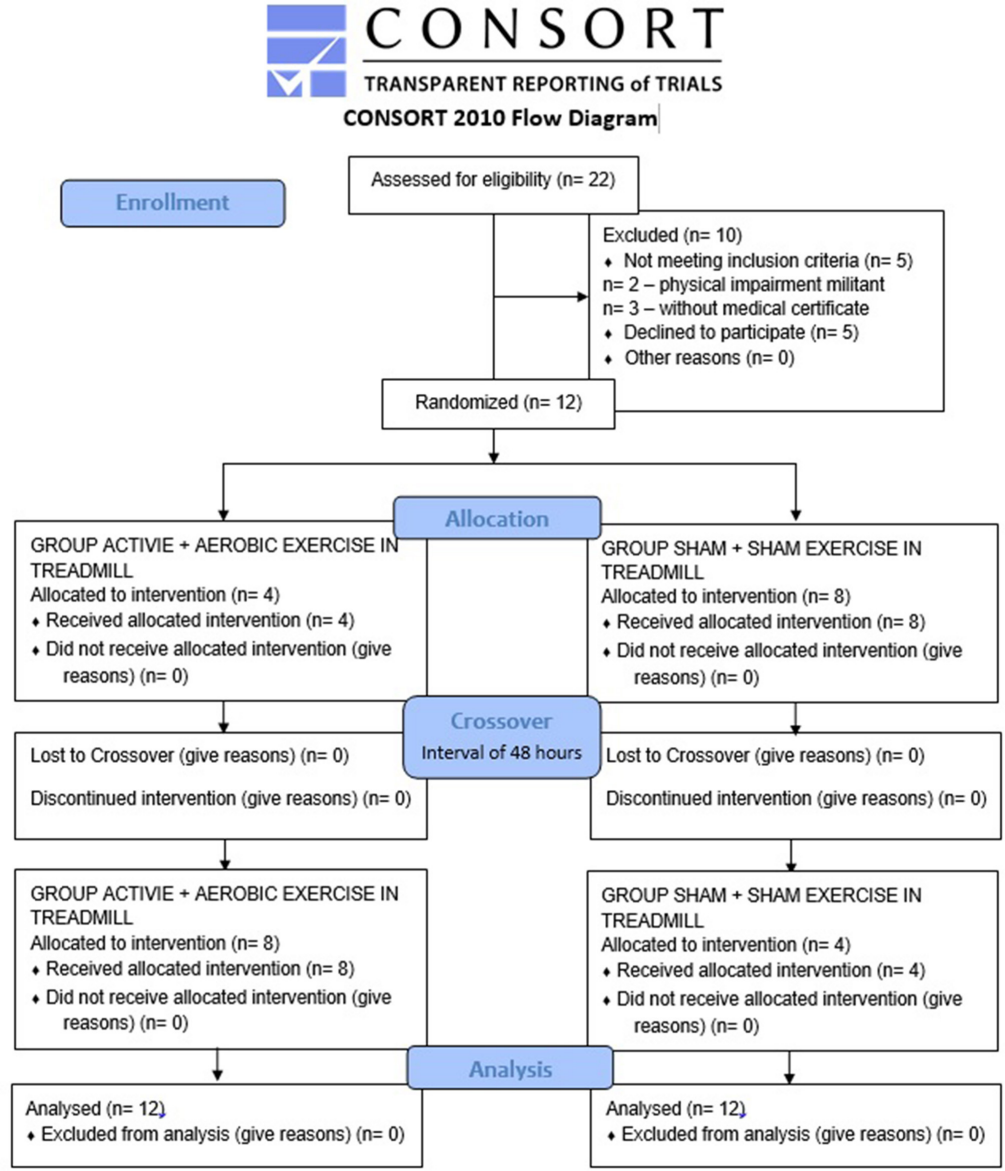
Flowchart of the study, followed CONSORT.

The patients were submitted to two intervention protocols crossover (active tDCS and sham tDCS) (Figure 1):

1. Evaluation of basal HRV (15 min), followed by active direct current transcranial stimulation (tDCS) (20 min). Immediately at tDCS, 15 min of HRV assessment followed by 20 min of treadmill walking, and finally another 15 min of HRV during exercise recovery.
2. Evaluation of basal HRV (15 min), followed by sham direct current transcranial stimulation (tDCS) (20 min). Immediately at tDCS, 15 min of HRV assessment followed by 20 min of treadmill walking, and finally another 15 min of HRV during exercise recovery.

Protocols (active tDCS or sham tDCS) were randomized to the first day of treatment only. On the second day (after 48 h) (17), the crossover was performed to be different from the first day.

Randomization was performed by lottery with brown, sealed envelopes to ensure reliability. The contents of each envelope determined which protocol the patient would initiate.

## Transcranial Direct Current Stimulation (tDCS)

The investigators responsible for the evaluation procedures and the patients were blinded to the treatment being applied (active tDCS or sham tDCS).

The patient was seated to receive the stimulus. Stimulation was performed with the NeuroConn tDCS DC-Stimulator Plus device using two 5 × 5 cm^2^ (non-metallic) surface electrodes, wrapped in a moist saline sponge with a 2 mA current for 20 min before exercise in running machine.

The anode electrode was positioned over the left temporal cortex (T3) and the cathode electrode over the middle deltoid muscle contralateral to the anode. In placebo stimulation, all lead positioning procedures were performed equally to the active tDCS procedure, but the stimulator was maintained on for 30 seconds (according to the fake device program). The patient was informed that he would feel a slight initial tingling, but would reduce, disappear or stay for 20 min after application. Thus, the patients had the initial feeling, but did not receive any stimulus in the remaining time. This procedure is a valid form of control in transcranial direct current stimulation studies (18).

## Acute Aerobic Exercise on the Treadmill

After 20 min of tDCS in a sitting position, the patients were positioned standing on the treadmill. To avoid possible falls, at least two physical therapists followed the exercise next to the patient. Cardiorespiratory parameters were monitored, such as heart rate, blood pressure, and oxygen saturation. Should these parameters be changed that put them at risk or if they expressed an interest in withdrawing from intervention, this would be immediately discontinued.

The velocity and inclination of each individual were determined by a previous exercise stress test using the modified Harbor protocol (14). Speed was kept constant and comfortable as determined by the individual and the treadmill inclination was increased by 2.5% every 2 min (19) until the patient reached 60–70% of the reserve heart rate (20). The inclination, therefore, was calculated to 60–80% of the maximum obtained in the ergometric test.

The electrocardiographic trace was acquired by (CPX Ultima, MedGraphics); heart rate (HR), oxygen saturation (SpO_2_) and blood pressure (BPA) were continuously recorded every 2 min of exercise. Dyspnea scores and perception of lower limb fatigue were measured at rest and immediately after an exercise session by the modified Borg scale (21).

## Evaluation of Heart Rate Variability (HRV) and Variability of Systolic Blood Pressure (VSBP), Obtained by Finometer Equipment

HRV and VSBP were collected in three moments (basal, immediately after the tDCS and after the exercise on the treadmill, during the exercise recovery). The data HRV were evaluated immediately after exercise for 15 min (during the exercise recovery). For this, the patient was positioned in seated and told not to move or speak.

For this, a Finometer hemodynamic meter (Finomiter® Pro/Finapres Medical Systems incorporates Modelflow technology) was used. It consists of equipment used to continuously monitor non-invasive cardiac pressure, which uses the technique of digital infrared photoplethysmography. For this procedure, an inflatable cuff is used around the middle finger phalanx.

Hemodynamic variables such as systolic (SBP), diastolic (DBP), mean (MBP), blood pressure (BP), cardiac output (CD) and total peripheral resistance (RPF) were assessed by beating and recorded in BeatScope software., based on values derived from the blood pressure curve and information on age, gender, weight, and height.

The data collection of blood pressure and its derivations such as the intervals R-R, observing the waves of high frequency (HF), low frequency (LF), and the low frequency interrelation with high frequency (HF/LF), were collected continuously in the basal periods and during the recovery from acute exercise for 15 min.

### Data Analysis of HRV and VSBP

The HRV analysis followed the Task Force's short-term spectral analysis in the time and frequency domain (22).

The cardiac modulation evaluation was performed by the register of interval R-R (ms) with the software *BeatScope*. The data were converted and stored in *Excel* files, used posteriorly for spectral analysis. A verification by visual inspection was performed by software *CardioSeries*, to identify and/or correct some premature or affected ectopic beats, using the linear interpolation to remove undesirable transients that alter the signal stationarity. The variations of Pulse Interval (PI) were evaluated in the time domain and the frequency domain by the linear method.

The linear methods were analyzed in the time domain and the frequency domain. Therefore, each RR interval was measured during a determined time interval, as described in the heart rate variability, calculating the floating transducer indexes of cardiac cycles.

RR intervals were collected in three periods: pretreatment (basal condition) for 15 min, immediately after tDCS application was completed (for 15 min) and during recovery from treadmill exercise (15 min).

In all three periods, the patient remained seated, without speaking or moving. Only 5 min of each patient's data were used for visual inspection by CardioSeries software to identify and/or correct some premature ectopic beats or artifacts, using linear interpolation to remove unwanted transients that could alter the signal stationarity of these patients analyzes in each period.

The indexes obtained by the determination of RR intervals by the time domain were the average of pulse interval RR through the absolute Variance and RMSSD that is the square root of the squared mean of the difference between the adjacent normal RR interval, in a time interval expressed in ms^2^ and is better correlated with the vagal modulation. But by the frequency domain, data were analyzed were through the high band (0.15 and 0.4 Hz), and low frequency (0.04 and 0.15 Hz) in its absolute values, and the sympathetic vagal balance.

## Statistical Analysis

The data were analyzed in the statistic program, *SPSSR* version 17.0 according to the distributed nature of variables, the central tendency measures, and the dispersion to be used as the averages and standard-deviation (parametric) or median and interquartile interval (non-parametric). HRV and BPV data are presented as mean ± SD or median and interquartile interval. For comparison between the applications, parametric tests (ANOVA of two repeated measure ways) and for the none-parametric tests were used (Friedman followed by the post-test Wilcoxon). *P* < 0.05 was considered statistically significant.

## Outcomes

Twenty-two patients were selected, 10 were excluded (two due to physical impairment that prevented them from performing the exercise, three for not having medical authorization for exercise testing and five for dropout (this dropout was reported for different reasons, such as disliking the exercise test (two patients), disliking having to stand still and not talking during HRV analysis (two patients), and unable to return to another assessment (Crossover) (one patient), thus constituting a sample of 12 patients.

To describe the demographic data of the patients, data were collected on age (years and months), topographic diagnosis of stroke, time of injury, classification (hemorrhagic or ischemic), affected hemisphere, weight (kg), height (cm), body mass index (BMI–kg/m^2^), blood pressure (BP), motor classification according to Fulg Meyer (mild, moderate, striking and severe).

All patients were instructed to appear always at 2:00 p.m. on assessment days, to avoid the influence of the circadian cycle. The prior recommendations were: continue with the use of medications on the respective hours, eat light diet on the test days, abstain from caffeine and ingestion of alcoholic beverages and/or smoking, avoid moderate or excessive effort on the previous day and the test day. The study procedures were performed in 6 moments.

The sample size was calculated using the mean of the three periods (baseline, post tDCS, and exercise recovery) of five individuals from the active tDCS group and five individuals from the tDCS sham group (crossover) from the pilot study.

To make the total average of the standard deviation, the sum of all deviations was calculated and divided by 6, which represents the total number of periods of the two groups.

To calculate the effect size (0.2453323), these values were entered in the G ^*^ Power 3.1.9.2 program and applied *F* tests, with a repeated-measures ANOVA statistical test between two factors with an α = 0.05, β = 0.80, and sample correlation of 0.5 giving an *n* = 90.

The clinic-demographic characteristics of patients are presented in Table 1.

**Table 1:** Descriptive characteristics of the sample.

**Variables**	***N* = 12**
Age (year)	59 ± 7.00
Gender (F/M)	12 (4/8)
Injury in right hemisphere	5 (41.7%)
Injury in left hemisphere	7 (58.3%)
Injury time (months)	65.45 ± 54.86
Ischemic stroke	9 (75%)
Hemorrhagic stroke	3 (25%)
Use of blockers	6 (50%)
Use of diuretics	2 (17%)
Use of calcium channel blockers	3 (25%)
Use of ECA inhibitors	2 (17%)
Use of angiotensin receiver blockers	6 (50%)
Use of type 2 diabetes medication	3 (25%)
Use of medication for cholesterol	3 (25%)
Use of medication for arthrosis	1 (8%)
Use of medication for spasticity	1 (8%)
*FulgMeyer*	77.27 ± 20.36
*FulgMeyer MMII*	25.58 ± 3.48
Light	3
Moderate	1
Marking	6
Severe	2
MEEM	24.33 ± 3.60
BMI (kg/m^2^)	26.52 ± 3.47
Weight (kg)	72.25 ± 11.03
Height (cm)	165 ± 9.92
HR basal (bpm)	71.42 ± 10.70
SBP basal (mmHg)	130 ± 8.42
DBP basal (mmHg)	76 ± 9.30
Comorbidities:	17
Diabetes	3
Hypertension	10
Positive HIV	1
Cholesterol	3

*Data expressed in average ± DP and frequency and percentage (%);F, Female; M, Male; MEEM, Mini exam of mental state, BMI, Body mass index; HR, Heart Rate; SBP, Systolic Blood Pressure; DBP, Diastolic Blood Pressure*.

Table 1 presents the demographic data for patients evaluated. The average body mass index (BMI) was 26.52 ± 3.47 kg/m^2^ with the average weight 72.25 ± 11.03 kg and an average height of 165 ± 9.92 cm, showing that the patients of the sample were overweight.

The heart rate average in rest was in (71 ± 10.70 bpm) within the normality and the SBP (130 ± 8.42 mmHg) and DBP (76 ± 9.30 mmHg) indicating stage 1 hypertension. All hypertense patients (83%) took medication for arterial hypertension, showing, therefore, a resistance hypertension degree.

Metabolic deficits such as diabetes or thyroid dysfunction were also not exclusion criteria, and of the 12 subjects participating in the study, three had drug-controlled type two diabetes.

All patients in the sample were overweight, as can be seen in Table 1, but after analyzing the baseline data between the groups, no differences were observed between the means as shown in Table 2.

**Table 2:** Values of HRV and VSBP of hemiparetic patients due to stroke in basal conditions.

**Groups** **(1 or 2)**	**Variance (ms)**	**RMSSD (ms)**	**HF abs (ms^**2**^)**	**LF abs (ms^**2**^)**	**LF/HF**	**Variance (mmHg)**	**LF (mmHg^**2**^)**	**α-LF**
Group 1	695.86 ± 326.28	17.89 ± 7.65	84.75 (13.84–157.03)	175.93 ± 119.34	2.29 ± 1.41	30.10 (19.82–45.96)	5.12 (2.82–10.66)	1.74 (0.94–2.20)
Group 2	852.24 ± 336.39	19.57 ± 8.28	135.81 (60.39–236.61)	213.56 ± 142.91	1.64 ± 1.10	26.04 (19.75–42.58)	5.61 (3.18–9.73)	1.73 (1.39–2.86)
Difference of averages	−0.89	−0.23	–	−1.45	0.94	–	–	–
IC 95%	(−416.17 to 181.32)	(−7.25 to 5.90)	–	(−190.58 to 40.04)	(−0.98 to 2.27)	–	–	–
P	0.40	0.82	0.51	0.18	0.38	0.48	0.58	0.39
Z	–	–	−0.65	–	–	−0.71	−0.55	−0.87

Variance, Total variability; RMSSD, Square root of the square mean of the difference between adjacent normal RR intervals; HF, High frequency band; LF, Low frequency band; abs, absolute; LF/HF, Balance sympathovagal; ms^2^, milliseconds to square; mmHg^2^, millimeters of mercury to square; α-LF, Low frequency alpha index; Mean ± Standard Deviation; Median (minimum-maximum); ^*^p < 0.05.

Table 2 presents the results of Heart Rate Variability (HRV) and Variability of Systolic Blood Pressure (VSBP) in basal conditions (pre active tDCS and sham tDCS) of hemiparetic patients due to stroke.

The result presented in Table 2 demonstrates that there was no difference in HRV and VSBP of patients due to stroke, in the basal condition of the two protocols. These data demonstrates that the patients found themselves in the same pre-test conditions.

In Table 3 are presented the resulted of VSBP in time and frequency domain in the periods basal, pos-tDCS and during the exercise recovery in the treadmill of Group 1 and 2.

**Table 3:** Values of VSBP of hemiparetic patients, in time and frequency domain, in the period's basal, post-tDCS, and post-exercise, of Groups 1 and 2.

**Groups (1 or 2)**	**Variance (mmHg)**	**LF (mmHg^**2**^)**	**α-LF**
Group 1 basal	30.10 (19.82–45.96)	5.12 (2.82–10.66)	1.74 (0.94–2.20)
Group 2 basal	26.04 (19.75–42.58)	5.61 (3.18–9.73)	1.73 (1.39–2.86)
Group 1 post-tDCS	33.07 (18.63–48.83)	9.81 (2.91–12.43)	1.54 (0.99–2.65)
Group 2 post-tDCS	35.86 (26.70–43.82)	8.82 (4.04–12.95)	1.94 (1.09–2.48)
Group 1 post-exercise	30.71 (25.75–41.70)	5.76 (4.27–10.71)	1.18 (0.63–1.88)
Group 2 post-exercise	31.25 (19.12–45.78)	5.76 (4.27–10.71)	1.45 (0.70–1.66)
P (Group 1)	0.78	0.78	0.74
P (Group 2)	0.53	0.37	0.53
Z	–	–	–

*Variance, Total variability; LF, Low frequency band; mmHg^2^, Millimeters of mercury to square; α-LF, Low frequency alpha index; Median (minimum-maximum)*.

The results of Table 3 show that there was no difference between the active groups and sham (1 and 2) in VSBP, in both the time domain and frequency domain in all periods analyzed.

Table 4 is presented the results of HRV in time and frequency domain in periods basal, pos-tDCS, and during the recovery from acute aerobic exercise post-exercise of Group 1 and 2.

**Table 4:** Values of HRV in time and frequency domain between pre and post-tDCS periods and post-exercise, of Groups 1 and 2 (active and sham).

**Groups (1 or 2)**	**Variance (ms)**	**RMSSD (ms)**	**HF abs (ms^**2**^)**	**LF abs (ms^**2**^)**
Group 1 basal	695.86 ± 326.28^*^	17.89 ± 7.65^*^	84.75 (13.84–157.03)^*^	175.93 ± 119.34^*^
Group 2 basal	852.24 ± 336.39^*^	19.57 ± 8.28	135.81 (60.39–236.61)	213.56 ± 142.91
Group 1 post-tDCS	1104.89 ± 95.15^*^	21.99 ± 10.75^*^	172.13 (50.30–366.73)^*^	329.90 ± 233.93^*^
Group 2 post-tDCS	1475.80 ± 632.90^*^	22.18 ± 9.08	146.01 (89.99–415.33)^*^	355.90 ± 249.83^*^
Group 1 post-exercise	751.50 ± 368.07	16.60 ± 5.70	65.01 (17.73–127.20)^*^	152.00 ± 122.65^*^
Group 2 post-exercise	852.81 ± 559.16^*^	16.18 ± 6.72	74.14 (21.07–139.18)^*^	168.00 ± 123.15^*^
Difference of Averages pre-post tDCS active	−409.02^*^	−4.10^*^	–	−165.59^*^
Difference of averages pre-post tDCS sham	−623.56^*^	−2.61	–	−142.34
Difference of averages basal—post-exercise active	−55.64	1.29^*^	–	2.39
Difference of averages basal—post-exercise sham	−0.57	3.38	–	45.50
Difference of averages post-tDCS–post-exercise active	353.38	5.39	–	167.98^*^
Difference of averages post-tDCS—post-exercise sham	622.99^*^	6.00	–	187.84^*^
IC 95% pre-post tDCS active	(−813.24 to −4.81)	(−7.85 to −0.34)	–	(−323.66 to −7.53)
IC 95% pre-post tDCS sham	(−1047.50 to −199.62)	(−6.54 to 1.31)	–	(−300.41 to 15.73)
IC 95% basal—post-exercise active	(−505.21 to 393.92)	(−4.76 to 7.35)	–	(−131.74 to 136.51)
IC 95% basal—post-exercise sham	(−472.07 to 470.94)	(−2.94 to 9.71)	–	(−88.63 to 179.63)
IC 95% post-tDCS—post-exercise active	(−126.58 to 833.30)	(−2.04 to 12.81)	–	(4.58–331.40)
IC 95% post-tDCS—post-exercise sham	(119.60–1126.38)	(−1.76 to 13.75)	–	(24.44–351.24)
Z pre-post tDCS active	–	–	−2.50^*^	–
Z pre-post tDCS sham	–	–	−1.72	–
Z basal—post-exercise active	–	–	−0.46	–
Z basal—post-exercise sham	–	–	−1.84	–
Z post-tDCS—post-exercise active	–	–	−2.50^*^	–
Z post-tDCS—post-exercise sham	–	–	−2.43^*^	–

tDCS, Transcranial Direct Current Stimulation; Variance, Total variability; RMSSD, Square root of the square mean of the difference between adjacent normal RR intervals; HF, High-frequency band; LF, Low-frequency band; abs, absolute; ms^2^, milliseconds to square; Mean ± Standard Deviation; Median (Minimum-Maximum);

* *p < 0.05 vs. adjusted for several comparisons of table: Bonferroni in parametric data and Wilcoxon in non-parametric*.

The results of HRV in Table 4 demonstrate that there was no difference between the active tDCS groups and sham in the following variables: Total variability (Variance), Square root of the square mean of the difference between adjacent normal RR intervals (RMSSD); Low frequency band absolute (LF abs) High frequency band absolute (HF abs).

In the period's post, tDCS and post-exercise in the linear analysis of time-domain of HRV intra-Group were observed that the variance value in the period post active tDCS increased in respect to the basal values, but did not maintain this increase in the post-exercise period. However, in the analysis intra-Group in the period post tDCS sham is also observed an increase in the variance value in respect to the basal values; however, in the period post-exercise of the group sham, there was a significant reduction in respect to the basal values.

Moreover, in the analysis intra-Groups, it was observed that RMSSD after active tDCS increased significantly and reduced after the exercise recovery period, but not significantly. In tDCS sham Groups, there was no significant difference in RMSSD.

In the frequency domain, it was observed an increase of Low-frequency band after the application of tDCS active and a reduction of the low-frequency band in the period post-exercise. However, in Group tDCS sham is observed the same Standard of high and low-frequency bands of active Group.

The effect size of active tDCS on intragroup HRV between post-tDCS periods compared to baseline HRV was calculated. As a result, an average effect size of (*d* = 0.44) was observed. There was no effect of active tDCS on HRV during the recovery period compared to baseline HRV.

In Table 5 are presented the short term analysis (1 min) for RMSSD for post-exercise recovery.

**Table 5:** Result of HRV in the time domain of 1-min RMSSD Wave Analysis between the basal period, post-tDCS and post-Exercise of Groups 1 and 2 (active and sham).

	**Group 1**	**Group 2**
RMSSD (ms)-basal Period	16.33 ± 7.52	17.50 ± 8.78
RMSSD (ms)-Post-tDCS Period	17.83 ± 7.25	18.83 ± 8.00
RMSSD (ms)-Post-Exercise Period	12.92 ± 4.64	13.92 ± 6.54
Difference of averages basal and Post-tDCS	−1.50	−1.33
Difference of averages basal and Post-exercise	3.42	3.58
Difference of averages Post-tDCS and Post-exercise	4.92^*^	4.92^*^
IC95% lower–top basal and Post-tDCS	(−4.42/1.42)	(−4.25/1.59)
IC95% lower–top basal and Post-exercise	(−1.24/8.08)	(−1.08/8.24)
IC95% lower–top Post-tDCS and Post-exercise	(0.50/9.33)	(0.50/9.33)
*p*-Value of averages basal and Post-tDCS	0.591	0.749
*p*-Value of averages basal and Post-exercise	0.212	0.177
*p*-Value of averages Post-tDCS and Post-exercise	0.026	0.026

HRV, Heart Rate Variability; tDCS, Transcranial Direct Current Stimulation; RMSSD, Square root of the square mean of the difference between adjacent RR intervals; Mean Standard Deviation; Median (lower-top);

* *p < 0.05 adjusted for the various comparisons in the table; Bonferroni in the parametric data*.

Table 5 demonstrate that there was no difference between the active tDCS and sham groups. Intra-group post-exercise period, short-term linear analysis (1 min) for RMSSD demonstrated reduction of RMMSD in active and sham tDCS groups.

An analysis of the effects of tDCS by subgroups of left hemispheric lesions vs. right were generated (Table 6) and shows that both the active tDCS and sham tDCS group did not show differences in HRV between the injured hemisphere, regardless of the three periods (baseline, post-tDCS and exercise recovery).

**Table 6:** Difference between sides of injury (right and left hemisphere) in baseline heart rate variability, post-TDCS and during exercise recovery.

**HRV active or sham at baseline, post-tDCS and exercise recovery**	**Right hemisphere injury**	**Left hemisphere injury**	**95% Confidence Interval for Difference**	***F***	***p***
Variance active at baseline	666.16 ± 153.23	720.61 ± 139.88	(−523.78/414.88)	0.069	0.799
Variance sham at baseline	892.21 ± 158.31	812.27 ± 158.31	(−436.32/596.21)	0.128	0.730
Variance active at post-tDCS	1117.08 ± 233.35	1094.73 ± 213.02	(−692.40/737.11)	0.005	0.945
Variance sham at post-tDCS	1559.15 ± 297.30	1392.45 ± 297.30	(−802.85/1136.26)	0.157	0.702
Variance active at exercise recovery	885.38 ± 162.65	639.94 ± 148.48	(−252.76/743.63)	1.242	0.294
Variance sham at exercise recovery	1134.87 ± 224.63	570.75 ± 224.63	(−168.44/1296.68)	3.153	0.114
RMSSD active at baseline	16.74 ± 3.55	18.72 ± 3.00	(−12.35/8.39)	0.181	0.679
RMSSD sham at baseline	22.73 ± 3.63	16.93 ± 3.32	(−5.33/16.92)	1.386	0.269
RMSSD active at post-tDCS	21.30 ± 5.03	22.48 ± 4.25	(−15.87/13.50)	0.032	0.861
RMSSD sham at post-tDCS	25.71 ± 3.97	19.24 ± 3.63	(−5.71/18.64)	1.442	0.260
RMSSD active at exercise recovery	16.28 ± 2.67	16.83 ± 2.26	(−8.35/7.24)	0.025	0.877
RMSSD sham at exercise recovery	20.19 ± 2.60	12.84 ± 2.37	(−0.61/15.31)	4.363	0.066
LF active at baseline	161.78 ± 36.10	118.72 ± 32.95	(−67.52/153.63)	0.776	0.401
LF sham at baseline	181.58 ± 65.81	240.22 ± 60.07	(−260.21/142.92)	0.433	0.527
LF active at post-tDCS	306.38 ± 106.47	301.81 ± 97.19	(−321.54/330.68)	0.001	0.975
LF sham at post-tDCS	415.12 ± 114.70	306.56 ± 104.71	(−242.77/459.88)	0.489	0.502
LF active at exercise recovery	142.48 ± 59.28	130.43 ± 54.12	(−169.54/193.64)	0.023	0.884
LF sham at exercise recovery	216.42 ± 53.80	127.78 ± 49.11	(−76.14/253.43)	1.481	0.255
HF active at baseline	89.44 ± 51.25	122.00 ± 51.25	(−199.69/134.56)	0.202	0.665
HF sham at baseline	175.13 ± 53.31	131.10 ± 59.61	(−145.07/233.12)	0.303	0.599
HF active at post–tDCS	172.28 ± 72.71	221.11 ± 72.71	(−285.95/188.28)	0.226	0.648
HF sham at post-tDCS	234.59 ± 84.86	194.98 ± 94.88	(−261.41/340.62)	0.097	0.765
HF active at exercise recovery	100.86 ± 30.75	61.29 ± 30.75	(−60.72/139.87)	0.828	0.389
HF sham at exercise recovery	132.82 ± 38.29	43.86 ± 42.81	(−46.86/224.79)	2.399	0.165
LF/HF active at baseline	2.63 ± 0.67	1.59 ± 0.55	(−0.97/3.04)	1.428	0.266
LF/HF sham at baseline	1.82 ± 0.55	1.60 ± 0.45	(−1.43/1.88)	0.098	0.762
LF/HF active at post-tDCS	2.13 ± 0.88	2.90 ± 0.72	(−3.40/1.85)	0.466	0.514
LF/HF sham at post-tDCS	1.79 ± 0.35	1.62 ± 0.28	(−0.86/1.21)	0.146	0.712
LF/HF active at exercise recovery	1.43 ± 0.54	2.23 ± 0.44	(−2.40/0.80)	1.329	0.282
LF/HF sham at exercise recovery	1.89 ± 0.98	3.21 ± 0.80	(−4.23/1.59)	1.099	0.325

*tDCS, Transcranial Direct Current Stimulation; Variance, Total variability; RMSSD, Square root of the square mean of the difference between adjacent normal RR intervals; HF, High-frequency band; LF, Low-frequency band; LF/HF, (high frequency/low frequency) ratio; Mean ± Standard Deviation; adjusted for several comparisons of table: Bonferroni in parametric data*.

Table 7 presents the results after active and sham tDCS of treadmill exercise time performance in minutes (min), distance covered in kilometers (Km) and degree of effort of lower limb fatigue and respiratory exhaustion by the modified BORG.

**Table 7:** Values of performance in the treadmill, time, distance elapsed, and the fatigue stress degree of lower limbs and breathing exhaustions by *BORG* modified od patients due to stroke.

**Groups (1 or 2)**	**Time in the treadmill (min)**	**Distance traveled (Km)**	***BORG* modified final breathing**	***BORG* modified final MMII**
Group tDCS active (1)	14.17 ± 5.08	0.61 ± 0.31	3.17 ± 1.40	3.50 ± 1.78
Group tDCS sham (2)	12.83 ± 5.37	0.52 ± 0.27	3.12 ± 1.76	3.67 ± 1.82
Difference of averages	0.96	2.18	0.11	−0.45
IC 95%	(−1.73 to −0.001)	(0.20–4.40)	(−0.82 to 0.90)	(−0.97 to 0.64)
*P*	0.36	0.05	0.92	0.66

min, Minute; Km, Kilometer; BORG, Subjective perception scale of effort; MMII, Lower limbs; Mean ± Deviation-Standard, *p < 0.05.

Observed in Table 7 that the active Group presented a significant increase of 90 m in the distance traveled in comparison with Group sham and the permanency time on the treadmill was greater in active Group (1 min), but not significant.

No difference was observed between the active Groups and sham in the perception of effort.

## Discussion

Considering the increase in patients surviving stroke events, it is important to increase knowledge regarding the mechanisms involved in the progression of lesions, as well as possible strategies for the management of this population, reducing the possibility of relapse and sudden death and providing a better quality of daily life. In this study, we evaluated the effects of a single exercise session, associated or not with prior tDCS stimulation, on BPPV and HRV in stroke survivors. Our results demonstrated that: there was no difference in VSBP and HRV between groups compared to baseline data; there was a reduction in time domain RMSSD band exercise recovery (short period linear analysis) in the groups with and without tDCS; however, in intragroup analysis, parasympathetic modulation after active tDCS increased the RMSSD by 18% from baseline, and the post-tDCS active and post-exercise periods had a variation value above baseline and, finally, and Importantly, there was better physical performance, as assessed by the increased distance covered in the active tDCS group, suggesting a better prognosis.

The research was composed of patients of both sexes (Table 1), with 83% of the sample presenting drug-controlled hypertension, but not yet classified as stage 1 hypertension (23). Hypertension is an important risk factor for stroke patients and is aggravated by the aging of this population (24). As a characteristic, patients were also overweight, which increases cardiovascular risk due to aortic stiffness (23).

The results in Table 2 showed that baseline HRV and VSBP values between the active groups and time domain and frequency domain simulation showed no differences, which demonstrates that the patients were in the same baseline conditions. Therefore, we show that the 48-h interval period was sufficient to avoid the effect of therapy addition (active or farce tDCS), corroborating with Nitsche and Paulus (25), who report that a 48-h interval between single session protocols is sufficient. to avoid possible effects additions.

The values of band LF absolute, in basal condition, suggested that the sympathetic system is already increased, what is already mentioned in the literature by Constantinescu et al. (26), Sander and Klingelhöfer (27), and Orlandi et al. (28), that describes that an acute ischemic stroke causes an autonomic unbalance capable of increasing the sympathetic cardiac activity, reduce HRV, and increase the risks of cardiac arrhythmia. It is important to remind the limitation about LF band of HRV and LF/HF ratio, sympatho-vagal balance, to provide indexes of the sympathetic activity. In these sense, there are evidences that LF band of HRV is modulated by the sympathetic and the parasympathetic nervous systems, thus LF band does not an index of sympathetic activity and the rationale for use LF/HF ratio, despite used as a cardiac sympatho-vagal balance, can be questioned (29).

In Table 3, the results of VSBP shows that the band of LF absolute of SBP was reduced in the basal condition, presenting an approximate value of 5 mmHg^2^. The normal values, of healthy individuals, present a mean LF band value of SBP around 17 mmHg^2^. This data suggests an exacerbated reduction of vascular sympathetic modulation, characteristic of sympathetic hyperactivity situations (30).

In respect to the results of VSBP after active tDCS and tDCS sham, no differences were found between the Groups and in the evaluation moments. However, we believe that the vascular sympathetic modulation may be reestablished, at least in part, post active tDCS. That was observed by the value of LF (of SBP) that increased ~48% (despite not significant) in respect to the basal values. These results corroborate with the studies of immediate effects of anodic tDCS described by Cogiamania et al. (10) and Piccirillo et al. (31) about the autonomic modulation. However, this was demonstrated in healthy individuals, reporting that the techniques no invasive of brain stimulation that effectively modulate the human cortical function may influence the blood pressure.

Piccirillo et al. (31) observed an improvement of temporal dispersion of myocardium, reduction in sympathetic sinus control, and increased vagal modulation in healthy elderly, after the single application of tDCS for 15 min, intensity 2 mA, over the left temporal area (T3) associated to breathing exercises. This differentiation of results observed in our study may have occurred for being a different population, and that already presents an autonomic modulation alteration, as described by Dutsch et al. (8) that report that independent of the ischemia side, the patient's post sever stroke present a parasympathetic cardiac deficit.

In respect to HRV (Table 4), our results demonstrate that there were no differences between the variables analyzed between the Groups active tDCS and sham, both post tDCS as well as during the exercise recovery. But, in the analysis of variables between the moment's basal, post tDCS and exercise recovery of each Group, a significant increase was observed of IP Variance in the time domain, between the moments basal and post tDCS in two Groups. However, only in Group sham, the post-exercise values were significantly reduced, demonstrating a domain of sympathetic activation and reduction of vagal modulation, already expected after an acute physical activity. The fact that there was no significant reduction of variance, that expresses the total variability after active tDCS, leads us to suggest that tDCS could maintain a greater parasympathetic modulation post-acute exercise and, therefore, a possible sympathetic regulation by a parasympathetic activation.

We also observed an increase in the RMSSD variable only in the Group active tDCS, showing a possible parasympathetic modulation by tDCS. These findings corroborate with the findings of de Paula et al. (32) that compare HRV and baroreflex sensitivity to aerobic resistance to acute exercise, on men with autonomic dysfunction, showing an increase of sympathovagal balance and a late standard of recovery of baroreflex sensitivity, what delays an abrupt sympathetic reduction.

In the HVR frequency domain of linear mode, still in Table 4, we have shown an increase in LF band in its absolute values only in Group active tDCS after stimulation. Furthermore, after acute exercise, was observed a reduction in both Groups, active tDCS, and sham. This reduction is unexpected, being a normal reaction after acute stress from exercise (33). The absolute HF variable shows a significant reduction after acute exercise in both groups, but only in Group active tDCS, there was an increase of this band after active tDCS, showing a possible action of tDCS in the cardiac parasympathetic modulation.

Another important data observed in this study was a significant increase of the distance traveled on the treadmill after active tDCS when compared to the tDCS sham, results that corroborate with the findings Okano et al. (9) and CICCONE et al. (34) that observed the increase of performance in healthy individuals when compared to Group sham.

The literature has demonstrated that tDCS may modify the autonomic responses in healthy individuals Vandermeeren et al. (35) and Clancy et al. (36), in healthy athletes Montenegro et al. (6) and Okano et al. (9). This is the first study that has as purpose to assess the effects of tDCS over the left temporal lobe, in HRV in individuals with stroke sequel. The region where the electrodes were placed in our study differ from Vandermeeren et al. (35) that stimulate frontal cortex, and the reference electrode was extracephalic. Clancy et al. (36) stimulate the primary motor cortex (M1) of none dominant hemisphere placing anode in C3 or C4 (international system 10/20 of EEG) and the cathode electrode over the supraorbital area contralateral to the anode electrode; Montenegro et al. (6) and Okano et al. (9) stimulate left temporal cortex (T3), and the cathode electrode was placed in the supraorbital area (Fp2) contralateral to anode electrode.

We opted to stimulate T3 to be the area closest to the insular cortex, responsible for autonomic control (37) and used by Montenegro et al. (6) and Okano et al. (9) with positive results in sympathetic modulation, observed by reduction and stability of heart rate of healthy athletes. These athletes performed better during a maximum incremental test, being 4% more efficient than their control group.

We performed more one analysis to see if the side of the lesion would affect our results (Table 6), since tDCS stimulation was only applied to the left temporal cortex, regardless of the side of the lesion, and the results showed no difference between groups, corroborating with Korpelainen et al. (38); Tokgözoglu et al. (39); Harms et al. (40); Constantinescu et al. (26) who report that neurovascular diseases such as ischemic stroke present changes in autonomic modulation with sympathetic hyperactivity, regardless of stroke lateralization.

Regarding the positioning of the reference electrode (cathode) we opted for the extracephalic (deltoid) positioning once according to Noetscher et al. (41) and removing the influence of cathode electrode action on the frontal cortex. Vandermeeren et al. (35), when studying the use of tDCS in the frontal cortex with an extracephalic reference electrode, under continuous cardiorespiratory monitoring and sympathovagal balance of healthy subjects, observed that subjects had a progressive reduction in respiratory rate, a constant increase in blood pressure over time, in which HR remained stable. However, regarding HRV, there was a trend of progressive change in sympathovagal balance, benefiting sympathetic tone, even when there is no significant difference between anode groups and cathode when compared with the tDCS sham, but proving to be a safe fit in healthy humans. Clancy et al. (36) observed autonomic modulation after active tDCS in M1 of healthy individuals.

There were no analyzes of all these data in our study regarding age. However, studies show that age alone is a cardiovascular risk factor (42). Aging promotes the reduction of vagal tone, favoring sympathetic predominance (42, 43).

Bonnemeier et al. (44) evaluated healthy individuals between 27 and 57 years old to verify the relationship of age with all HRV parameters and demonstrated that the parameters with the highest correlation with age were SDNNi, SNN50, and rMSSD; noting a decrease in vagal control as they get older, being more prominent among women.

In addition, systolic and diastolic blood pressure tends to increase with increasing age, especially in women, along with changes in peripheral and central mechanisms that help maintain adequate brain flow (45).

However, it should be considered that in our study we evaluated fewer women than men who would have more influence on age. Moreover, the presence of established hypertension and drug use in all research subjects, besides being stroke survivors, certainly have a greater impact on cardiovascular autonomic modulation than age. However, this can be considered a limiting factor in our study.

## Final Considerations

This study supplies evidence that a single stimulation with tDCS over the left temporal cortex (T3) of hemiparetic patients due to stroke may improve the cardiac parasympathetic response (RMSSD and HF band of IP). However, significant differences were not observed in the other parameters studied. These results may provide the base for future perspectives of researches about the autonomic modulation after tDCS in stroke.

This study is one of the first studies purposes for evaluating the effects of tDCS over the autonomic modulation in hemiparetic patients due to stroke. Therefore, such opens a path for the betterment of a protocol purpose of verifying the best positioning of electrodes, best stimulation moment, as well as the best aerobic training protocol to be associated to electric stimulation over the cerebral cortex. We consider being important the continuity of the researches in this field for the improvement of knowledge and development of this treatment method considering the advantages listed.

## Study Limitations

We consider some topics as limitations of our study:

Difficulty in recruiting patients due to their limited mobility;

Some patients had insecurity to walk on the treadmill. This fear may have influenced the increase in stress and, consequently, a greater parasympathetic action;

Absence of other neurophysiological evaluations such as electroencephalogram or lipid analysis, to better understand the effects of treatment.

Absence of complementary exams for an accurate diagnosis regarding the location and extent of the lesion. Patients with metabolic deficits such as diabetes or thyroid dysfunction were not excluded.

Study data were not analyzed by age. All patients were overweight.

## Data Availability Statement

TRequests for data can be directed to Glauber Heinz, glauber.heinz@hotmail.com.

## Ethics Statement

The studies involving human participants were reviewed and approved by the proposed study complies with the principles of the Declaration of Helsinki and the Regulating Norms and Directives for Research Involving Human Subjects formulated by the Brazilian National Health Council established in October 1996. The study received approval from the ethics committee of Universidade Nove de Julho (São Paulo, Brazil) under protocol CAAE: 57314716.7.0000.5511 and is registered with the International Registry of Clinical Trials (ClinicalTrials.gov–NCT02956096). The participating institutions have provided a declaration of participation and will be allowed to abandon the study at any time with no negative repercussions. The patients/participants provided their written informed consent to participate in this study.

## Author Contributions

GH and FC conceived and planned the theory and drafted the manuscript. GH and MS carried out the experiment. KD, SD, AV, FD, and JC helped supervise the project. GH, MS, and FC conceived the original idea. GH, MS, AV, and FD developed the theoretical formalism and participated in the design of the study. SD participated the performed the statistical analysis. FC oriented the project and the authors KD and SD coordinated the project. All the authors have made substantive contributions to the manuscript and assume full responsibility for its content, conceived of the study, participated in its design and coordination and helped to draft the manuscript, read and approved the final manuscript. All authors agree to be accountable for the content of the work.

### Conflict of Interest

The authors declare that the research was conducted in the absence of any commercial or financial relationships that could be construed as a potential conflict of interest.
